# Medical Journal Publishing: Continued struggle is essential to Achieve and maintain standards

**DOI:** 10.12669/pjms.36.4.2685

**Published:** 2020

**Authors:** Shaukat Ali Jawaid

During the Year 2019, Pakistan Journal of Medical Sciences received a total of one thousand four hundred nineteen manuscripts. Out of this, three hundred twenty five were published, nine were rejected due to plagiarism, thirty one were withdrawn by the authors as they were keen to get it published immediately while the remaining were not accepted for further processing during initial screening for various reasons. Table-I. During the same period thirty manuscripts were accepted for fast track processing, twenty five such requests were rejected. Fast track processing facility was started to help those authors who either had to appear in some examination or had to submit their Thesis for PhD or M. Phil and it was essential to get a paper published before that. However, it was also observed that an attempt was made by some authors to misuse this facility as their promotion was due or they just wanted to get their manuscripts published early. Since our objective is to help facilitate authors to publish their research work and not to make money, as a policy it was decided that those who wish to get their papers processed on fast track must first ask for permission, specify the reasons and if accepted only then they should opt for this and arrange fast track processing fee. The idea was to discourage authors to make use of this facility unless it was extremely essential. We do Publication Audit regularly every year as it helps to identify the strength and weaknesses that is very helpful in improving the contents as well as standard of the journal.^1^-^3^

**Table-I:** PJMS manuscripts statistics of 2019 at a Glance.

Total Published Articles:	325
Total Rejected Articles:	981
Total rejected article on plagiarism:	9
Total Withdraw Articles:	31
Under Process:	73
Total Received Articles:	=SUM(ABOVE) 1419

**Fig.1:**
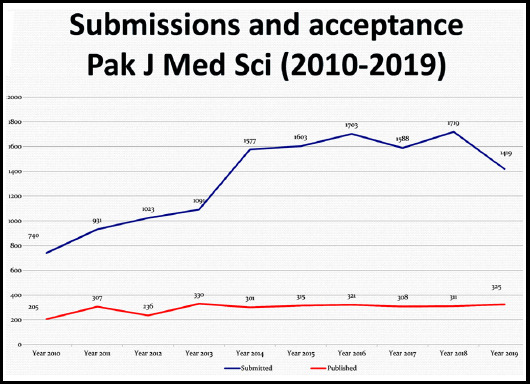


Yet another policy decision taken was that if the authors had any doubt about acceptance of their paper for further processing, they were asked to first share the abstract of their manuscript by e-mail and get an opinion whether we can process their manuscript or not and if they get a positive response, only then complete the other formalities of submission on the journal website with all the necessary documents like Letter of Undertaking of exclusive submission and Ethics Committee approval besides arranging processing fee. It not only helped the authors to save their money but also saved the time of the editorial team to reduce the number of submissions on the website for initial screening and further processing. That is one of the reasons that the graph of submission during the Year 2019 shows a little downward trend. Fig.1.

A detailed analysis of the submissions during the Year under review also showed that as usual more submissions from overseas were from countries like Turkey, China, and Saudi Arabia i.e. Three hundred thirty four, one hundred forty seven and one hundred thirty eight respectively. Table-II. Over the years the number of submissions from China, Iran has drastically reduced simply because we are more careful, selected only good quality manuscripts besides encouraging the authors to publish their manuscripts in their local journals. As such the number of manuscripts published from these countries since 2010-2019 has varied. Table-III.

**Table-II:** Country wise submissions during 2019.

Country	Total
Afghanistan	2
Albania	1
Algeria	1
Antarctica	1
Argentina	1
Armenia	1
Australia	1
Azerbaijan	1
Bangladesh	1
Brazil	2
China	147
Christmas Island	2
Cyprus	4
Egypt	10
India	7
Indonesia	55
Iran	53
Iraq	30
Italy	2
Jordan	4
Korea	9
Malaysia	14
Nigeria	10
Pakistan	559
Palestinian	2
Pitcairn	1
Romania	2
Russia	2
Rwanda	1
Saint Barthelemy	2
Saudi Arabia	138
Singapore	2
Swaziland	1
Thailand	1
Turkey	334
Ukraine	1
United Arab Emirates	1
United Kingdom	5
United States	5
Viet Nam	3

Grand Total	1419

**Table-III:** Manuscript published by Pak J Med Sci (2010 – 2019).

Country	2010	2011	2012	2013	2014	2015	2016	2017	2018	2019
Algeria							1			1
Australia							1	1	1	1
Bahrain	1			1	1					
Bangladesh	4	4	4		1	1	1		1	
Cameroon	1									
China	1	18	34	80	89	65	66	54	43	46
Cyprus			1	1					3	1
Fiji						1				
Germany				1						
India	2	1		1		1	1		1	1
Iran	64	78	63	70	14	12	11	10	3	8
Iraq	1	3	2	3	1		1		2	3
Japan								1		
Jordan	4		1							1
Kenya			1							
Korea	1	2	1	3	2	5	7	2	2	1
Kuwait	1		1							
Malaysia	1	9	3	9	7	10	6	1	1	
Nigeria	10	9			4	4	1			
Oman	1		1			1				
Pakistan	56	93	65	91	93	106	135	163	150	168
Palestine	2			3	1	2				
Philippines							1			
Poland					2	4				
Romania				1	1	3	1	1	1	
Saudi Arabia	11	6	16	17	19	32	23	21	24	32
Serbia								1	1	1
South Africa	3	2	1	6	2		1			
Sudan	1									
Taiwan		2	2	3						
Thailand									2	
Turkey	34	74	37	38	60	65	63	49	72	60
UAE	4	1	1			2		1	3	
Uganda			1							
UK	2	5	1	2		1	1	1	1	1
USA					4			2		

Total (36)	205	307	236	330	301	315	321	308	311	325

Total number of papers received from Pakistan during the Year 2019 was five hundred fifty nine while the number of papers published from Pakistan was one hundred sixty eight. Over the years there has been a progressive increase in the number of papers published from Pakistan because we wish to encourage more Pakistani authors to publish in local journals. While in the past the percentage of papers from Pakistan in each issue of Pakistan Journal of Medical Sciences used to just about 20%, now it has increased to almost 50%. We have also observed an appreciable improvement in the quality of manuscripts that we attract from the authors from Pakistan as well. Table-IV. As expected the largest number of manuscript published during the Year 2019 were original articles. Table-V. A further analysis of submission from Pakistan revealed that maximum number of manuscripts was submitted from Karachi followed by Lahore, Peshawar and Rawalpindi despite the fact that a large number of medical journals are also published from these cities but it is also a fact that more research work is also undertaken in these cities for various reasons. Table-VI. However, it was heartening to note that the journal attracted manuscripts from many small cities from all over the country as well which is a witness of increasing readership and popularity of the journal among the healthcare professionals.

**Table-IV:** Country Wise Manuscript Published in 2019.

Country	Jan-Feb 2019	Mar-April 2019	May-June 2019	July-Aug 2019	Sep-Oct 2019	Nov-Dec 2019	Grand Total
Algeria	--	--	--	--	1	--	1
Australia	--	--	--	--	1	--	1
China	8	8	9	6	10	5	46
Cyprus	--	--	1	--	--	--	1
India	--	--	1	--	--	--	1
Iran	1	2	2	1	1	1	8
Iraq	--	--	--	2	1	--	3
Jordan	--	--	--	--	--	1	1
Korea	--	1	--	--	--	--	1
Pakistan	31	26	23	28	31	29	168
Saudi Arabia	2	4	9	9	1	7	32
Serbia	--	--	--	1	--	--	1
Turkey	13	13	9	8	8	9	60
United Kingdom	--	--	1	--	--	--	1

Grand Total	55	54	55	55	54	52	325

**Table-V:** Category Wise Manuscript Published in 2019.

Category	Jan-Feb 2019	Mar-Apr 2019	May-Jun 2019	Jul-Aug 2019	Sep-Oct 2019	Nov-Dec 2019	Grand Total
Case Reports	--	2	1	--	1	2	6
Conference Proceedings	--	--	--	--	--	1	1
Correspondence	--	--	1		1	1	3
Editorial	1	--	--	2	--	--	3
Guest Editorial	--	--	1		--	--	1
Original Articles	52	48	51	51	51	46	299
Review Article	1	--	--	1	--	1	3
Short Communication	1	1	--	--	--	1	3
Special Communications	--	--	1	--	--	--	1
Systematic Review	--	3	--	1	1	--	5

Grand Total	55	54	55	55	54	52	325

**Table-VI:** City wise submissions from Pakistan during 2019.

Cities	Total
Abbottabad	1
Azad Kashmir	1
Bahawalpur	4
Chitral	1
D.I. Khan	1
Faisalabad	11
Gambt, Khairpur, Sindh	1
Gilgit	3
Gujranwala	4
Gujrat	10
Haripur	1
Hyderabad	6
Islamabad	59
Jamshoro	5
Karachi	207
Karak	1
Kasur	1
KhairpurMirs, Sindh	1
KharianCantt.	1
Kohat	1
Lahore	112
Larkana	1
Mansehra	1
Mardan	1
Mirpurkhas	1
Multan	11
Muzaffarabad	1
Nawabshah	2
Peshawar	42
Poonch, Azad Kashmir	2
Quetta	10
Rabwah, Punjab	1
Rahim Yar Khan	3
Raiwind, Punjab	1
Rawalakot, Azad Kashmir	2
Rawalpindi	26
Sargodha	5
Sialkot	5
Sukkur	1
Swat	2
Tando M. Khan	2
Wazirabad	1
Dera Ghazi Khan	3
Mirpur, Azad Kashmir	1
Shikarpur	1
Dera Ismail Khan	1

Grand Total	559

**Fig.2:**
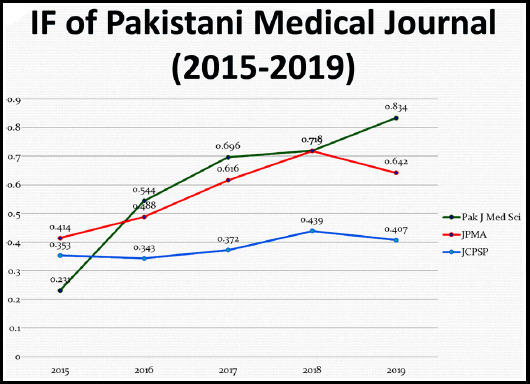


Since the objective of the Journal and its Editorial team is not just to accept, reject and publish papers but also teach and train the authors, Editorial team of Pakistan Journal of Medical Sciences has been actively participating in organizing as well as facilitating workshop on Scientific Writing, Peer Review and training courses on Journal publishing not only in Pakistan but in the EMRO region as well.^4^ Such academic activities were organized in Riyadh Saudi Arabia, Gulf countries as well in addition to pre-conference workshops at various medical institutions in collaboration with different professional specialty organizations. However, still there is lot of pressure from the authors and those whose manuscripts are not accepted for further processing or publication are not happy. It is this strict policy and peer review system that has helped us to retain the highest Impact Factor among the Pakistani biomedical journals for the last many years. Fig.2. However, the authors must realize that we have our own financial as well as human resource constraints. Hence, even if we entertain more manuscripts for further processing, it will take too long to get them published and the authors will have to wait much longer which they will neither like nor it will be in their interest. Hence, it is better if they submit their manuscripts to those journals which can process them early. Every author is keen to get their research work published as soon as possible but at times it is not possible. The solution lies in having more and more good quality peer reviewed biomedical journals.

Keeping the above in view, Pakistan Journal of Medical Sciences as a member of PAME in collaboration with some other distinguished editors has initiated a Certificate Course in Medical Editing at University of Health Sciences at Lahore. It is a joint project of PAME with UHS in which we have the support of many distinguished researchers and editors from Pakistan as well as overseas who have agreed to help and mentor these course candidates. Sixty candidates applied for the first course out of which thirty were selected, preference being given to those who were already affiliated with some journal. The first batch has completed the course and their final portfolio assessment and Viva Exam was scheduled on March 16^th^ 2020 which had to be postponed due to COVID19. We hope to organize this as soon as the situation improves and arrangement will be made to induct the second batch hopefully before August 2020. 5

Yet another decision we have taken is to publish two special issues every year exclusively of Case Reports. Though they usually have a low priority with us but many authors are keen to get their case reports published and it is also the ideal way to start publishing for many young authors. These issues will be only Online Publications and no print copies will be available. However, they will go through the routine initial screening, plagiarism check, peer review, will have DOI number and they will also be visible online through PubMed Central. Publication of some other special issues, thematic issues will also be considered. In view of the current COVID19 Pandemic, a special issue on Corona Virus has also been planned which will be published in the next two months. Submissions in this issue have been processed on fast track on complimentary basis just to help authors.

In short every effort is being made to help authors by following an author friendly policy to promote research culture, the art of medical writing and scientific publishing in the country. Numerous informative manuscripts which provide details about the manuscript processing system, peer review policy, guidelines for the authors following which they can minimize trauma to their submitted manuscripts are published from time to time all aimed at helping the authors. 6-7 However, it is extremely difficult to please everyone and our experience also shows that those who are more impatient and keen to get their manuscripts processed on fast track, the quality of most of these manuscripts is not so good. In a hurry, these authors even do not bother to carefully read and follow the instructions for authors on journal website which leads to enormous delay in processing of their manuscripts. It is in their own interest to read and follow these instructions in letter and spirit and also look at some of the published papers on our website before making their submissions.
